# Global Occupational Exposure to Blood and Body Fluids among Healthcare Workers: Systematic Review and Meta-Analysis

**DOI:** 10.1155/2022/5732046

**Published:** 2022-06-03

**Authors:** Dechasa Adare Mengistu, Gebisa Dirirsa, Elsai Mati, Dinku Mekbib Ayele, Kefelegn Bayu, Wegene Deriba, Fekade Ketema Alemu, Yohannes Mulugeta Demmu, Yohanis Alemeshet Asefa, Abraham Geremew

**Affiliations:** Department of Environmental Health, College of Health and Medical Science, Haramaya University, P.O. Box 235, Harar, Ethiopia

## Abstract

**Background:**

Occupational exposure to blood and body fluids has become a serious public health problem for healthcare workers and is a major risk for the transmission of various infections such as human immune-deficiency virus, hepatitis B virus, and hepatitis C virus. This systematic review and meta-analysis aims to determine the career time and previous one-year global pooled prevalence of occupational exposure to blood and body fluids among healthcare workers.

**Methods:**

For the review, the articles published in English were searched using the electronic databases (SCOPUS/Science Direct, PubMed, Web of Science, Google Scholar, CINAHL, MEDLINE, Cochrane Library, DOAJ, and MedNar) with a combination of Boolean logic operators (AND, OR, and NOT), Medical Subject Headings (MeSH), and keywords. A quality assessment was conducted to determine the relevance of the articles using JBI critical appraisal tools. Furthermore, several steps of assessment and evaluation were taken to select and analyze the relevant articles.

**Results:**

Of the 3912 articles identified through the electronic database search, 33 that met the inclusion criteria were included in the final analysis. The current study found that the global pooled prevalence of blood and body fluids among healthcare workers during career time and in the previous one year accounted for 56.6% (95% CI: 47.3, 65.4) and 39.0% (95% CI: 32.7, 45.7), respectively. Based on subgroup analysis by publication year, survey year, and World Health Organization regions, the highest prevalence of blood and body fluid exposure in the last 12 months was observed among articles published between 2004 and 2008 (66.3%), conducted between 2003 and 2008 (66.6%), and conducted in the Southeast Asia Region (46.9%). The highest career time prevalence was 60.6%, 71.0%, and 68.4% for articles published between 2015 and 2020, conducted between 2015 and 2019, and reported in the African region, respectively.

**Conclusion:**

The current study revealed a high prevalence of occupational exposure to blood and body fluids among healthcare workers and suggests the need to improve occupational health and safety services in healthcare systems globally.

## 1. Introduction

Occupational exposure to blood-borne pathogens as a result of contact with human blood and body fluids has become a serious health concern for healthcare workers (HCWs) globally [1]. Occupational exposure to blood and body fluids (BBFs) constitutes a risk of transmission of blood-borne pathogens, such as human immune-deficiency virus (HIV), hepatitis B virus (HBV), and hepatitis C virus (HCV) [2–5], and other blood-borne pathogens, including cytomegalovirus, herpes simplex virus, and parvovirus B19 [4]. Healthcare workers are at high risk of being infected with various occupational-related diseases as a result of exposure to blood-borne pathogens [1, 5, 6].

The risk of transmission of infection after exposure to infected blood is 0.3% times greater for human immunodeficiency virus-infected blood than for uninfected blood, while it is estimated to be up to 100 times greater for the hepatitis B virus and from 3 to 10% for the hepatitis C virus [7, 8]. Among the above infections (HBV, HCV, and HIV), only HBV had a vaccine until the time of this study [7].

According to the World Health Organization (WHO) report, about three million HCWs are exposed to blood-borne pathogens each year, of which 170,000 are exposed to HIV infections, 2 million to HBV infections, and 0.9 million to HCV infections [9]. Most of the time, healthcare providers get exposure through the splash of blood or other body fluids into the eyes, nose, or mouth or nonintact skin exposure, and percutaneous injury occurs as a result of a break in the skin caused by a needlestick or sharps contaminated with blood or body fluids [9].

Several studies, including systematic reviews and meta-analysis, have been conducted and published on the prevalence of BBFs among HCWs in different settings, such as at country or region levels. And also, a few studies reported the global prevalence of occupational exposure to needlestick injuries [10], the prevalence and device-related causes of needlestick injuries [11], percutaneous injury [1], and the prevalence of exposure to blood and body fluids in Africa [6].

However, there is no evidence regarding the global prevalence of blood and body fluids among healthcare workers. Therefore, this is the only study that provides a global prevalence of blood and body fluid exposure among healthcare workers, which can be used as evidence and input to reduce the burden of BBF exposure and may prompt the development of appropriate policies, systems, and processes. Furthermore, this systematic review and meta-analysis estimated the regional levels, last year, and career time prevalence of BBFs among HCWs.

## 2. Materials and Methods

### 2.1. Protocol Registration and Search Strategy

The research protocol was registered in the PROSPERO international prospective register of systematic reviews (CRD42017077201). The Preferred Reporting Items for Systematic Reviews and Meta-Analysis (PRISMA) guideline was used to perform this systematic review [12].

### 2.2. Eligibility Criteria

#### 2.2.1. Inclusion Criteria

The studies that met the following inclusion criteria were included in the systematic review and meta-analysis:

- Study population: healthcare workers regardless of their occupation
- Outcomes: study reporting quantitative outcomes (magnitude, frequency, rate, or prevalence of BBFs in lifetime and/or last year)
- Language: studies written in English
- Types of articles: peer-reviewed full text, original, and published articles
- Publication year: not specified (not limited)
- Study region or country: not specified (not limited)

#### 2.2.2. Exclusion Criteria

Studies that did not report 12 months or career time prevalence (such as 3 or/and 6 months) of BBFs, case reports, case series, review articles, surveillance data, reports, conference abstracts, personal opinions, articles written in non-English, high risk of bias articles, and studies not available in full texts were excluded from the current study.

### 2.3. Information Sources and Search Strategy

The articles were searched using ten electronic databases (SCOPUS/Science Direct, PubMed, Web of Science, Google Scholar, CINAHL, MEDLINE, Cochrane Library, DOAJ, and MedNar) using a combination of Boolean logic operators (AND, OR, and NOT), Medical Subject Headings (MeSH), and keywords, such as health professionals, healthcare workers, healthcare system, developing country, developed country, blood, blood and body fluids, and occupational exposure.

The articles were searched using a combination of Boolean logic operators (AND, OR, and NOT), Medical Subject Headings, and keywords. The following is a search term used in the initial search: “prevalence” [MeSH Terms] OR “prevalence” [All Fields]) AND ((“occupational” [MeSH Terms] OR “occupational” [All Fields], OR “work place” [All Fields] OR “work place” [MeSH]) AND ((“blood and body fluids” [MeSH Terms]] OR (“blood” [All Fields] AND “fluids” [All Fields]) OR “blood and splash” [All Fields]) OR “healthcare workers” [MeSH Terms] OR “healthcare” [All Fields] AND “workers” [All Fields]) OR “healthcare workers” [All Fields]) OR “health professional” [All Fields]) OR “health professional” [All Fields]) OR “health professional” [All Fields])” OR (“health” [All Fields] AND “provider” [All Fields]) OR “health provider” [All Fields])) AND (“developing country” [MeSH Terms] OR (“developing” [All Fields] AND “countries” [All Fields]) OR “developing countries” [All Fields]) OR “developed countries” [MeSH Terms] OR (“developed” [All Fields] AND “countries” [All Fields]) OR “developed countries” [All Fields])).

Then, all identified keywords and index terms were checked across the nine electronic databases included. Finally, searching the reference list of all identified articles for further articles was conducted.

### 2.4. Study Selection

The study selection process was performed using the PRISMA flowchart, indicating the number of articles included in the review and articles excluded from the study with reasons. Following the search for articles through selected electronic databases, duplicate studies were removed using the ENDNOTE software version X5 (Thomson Reuters, USA). The authors independently selected the articles based on the titles and abstracts by applying the inclusion criteria. Furthermore, the full text of the relevant articles was further read in detail and independently evaluated by the authors. Any disagreements made with respect to the inclusion of studies were resolved by consensus after discussion. Finally, studies that met the inclusion criteria were included in the systematic review and meta-analysis.

### 2.5. Data Extraction

The authors (DAM, GDG, EM, DMA, KB, WD, FKA, and YAA) independently extracted the data from the included articles. A predefined Microsoft Excel 2016 format was used to extract information from selected studies under the following headings: author; publication year; country of study; study design; primary outcomes such as prevalence or magnitude of exposure to BBFs and possible confounding factors considered. In general, all data are extracted from the eligible articles.

### 2.6. Quality Assessment

The selected articles were subjected to a rigorous independent assessment using a standardized critical assessment tool, the Joanna Briggs Institute (JBI) Critical Assessment Tools for prevalence studies [13]. These articles were then evaluated by the authors (DAM, GDG, YMD, YAA, and AG) to confirm their relevance to the study and the quality of the work.

The evaluation tools have the following nine evaluation criteria or parameters: (1) appropriate sampling frame; (2) proper sampling technique; (3) adequate sample size; (4) description of the study subject and setting description; (5) sufficient data analysis; (6) use of valid methods for identified conditions; (7) valid measurement for all participants; (8) use of appropriate statistical analysis; and (9) adequate response rate. Failure to satisfy each parameter was scored as 0, if not 1. The score was then given across all studies and graded as high (85% and above), moderate (60–85% score), or low quality (60% score). Disagreement made on what was to be extracted was solved by discussion after repeating the same procedures. The PRISMA guidelines protocol [12] was used to conduct the review.

### 2.7. Statistical Procedures and Data Analysis

The pooled prevalence of the BBFs was performed using Comprehensive Meta-Analysis (CMA) version 3.0 statistical software. A forest plot and a random-effects model were used to determine and visualize the pooled prevalence of the BBFs.

The Cochran *Q* test (*Q*) and *I* squared test (*I*^2^ statistics) were used to evaluate the heterogeneity between the included articles. *I*^2^ statistics is the proportion of variation in prevalence estimates due to genuine variation in prevalence [14, 15]. The level of heterogeneity was classified into four categories: no heterogeneity (0%), low (25–50%), moderate (50–75%), and high heterogeneity (greater than 75%) [16]. The random-effects model was used to analyze the data. Furthermore, subgroup analysis was conducted based on the year of publication, survey period (when the study was conducted), and study areas. Publication bias among the included studies was evaluated using funnel plots. A sensitivity analysis was done to determine differences in pooled effects by dropping studies that were found to influence the summary estimates.

## 3. Results

### 3.1. Study Selection

**Figure 1:**
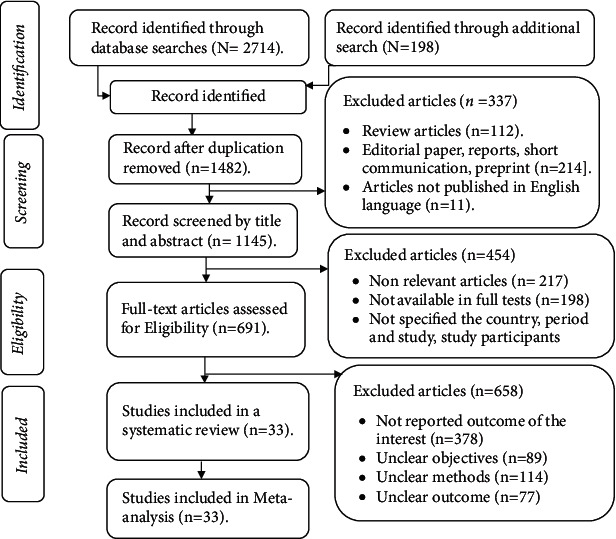
Study selection process of included articles for systematic review and meta-analysis, 2021.

A total of 2912 studies were retrieved from searches in selected electronic databases. Then, 1430 duplicate articles were excluded. Out of 1610 nonduplicated studies, 327 studies were excluded based on titles and abstracts. Furthermore, 1759 full-text studies were further assessed to determine their eligibility, of which 1724 studies were excluded. These articles were excluded as a result of not reporting the prevalence of blood and body fluids in their career time or last year; unclear objectives, unclear methods, not available in full text; nonhealthcare worker study participants; review articles; letters to the editor; brief reports; and written in a non-English language. Finally, 33 studies that met the inclusion criteria were included in the review (Figure 1).

### 3.2. Study Characteristics

This systematic review and meta-analysis included a total of 33 studies conducted on 54328 HCWs in 18 countries from 2003 to 2021. The sample size of included studies ranged from 64 to 33156 healthcare workers. Seventeen articles were conducted in developing countries. The highest prevalence of exposure to BBFs in the last year and career time was reported in China and Ethiopia, respectively. Among the included studies, 4 articles were conducted in Ethiopia [2, 17–19], 3 were conducted in South Africa [20–22], 3 were conducted in Serbia [23–25], 3 were conducted in Iran [26–28], 3 were conducted in China [29–31], 2 were conducted in Tanzania [32, 33], 2 were conducted in India [34, 35], 2 were conducted in United Arab Emirate [36, 37], 2 were conducted in Nigeria [38, 39],and 1 was conducted in each of Thailand [40], Kenya [41], Turkey [42], Lebanon [43], Bosnia and Herzegovina [44], Togo [45], Georgia [46], Croatia [47], and USA [48]. About three-quarters were conducted in hospitals (Table 1).

### 3.3. Prevalence of Blood and Body Fluids

This systematic review and meta-analysis was conducted using the Comprehensive Meta-Analysis (CMA) Version 3 statistical package (software) to determine the pooled prevalence of blood and body fluids among healthcare workers.

#### 3.3.1. Previous Last Year Prevalence of Exposure to Blood and Body Fluids

**Figure 2:**
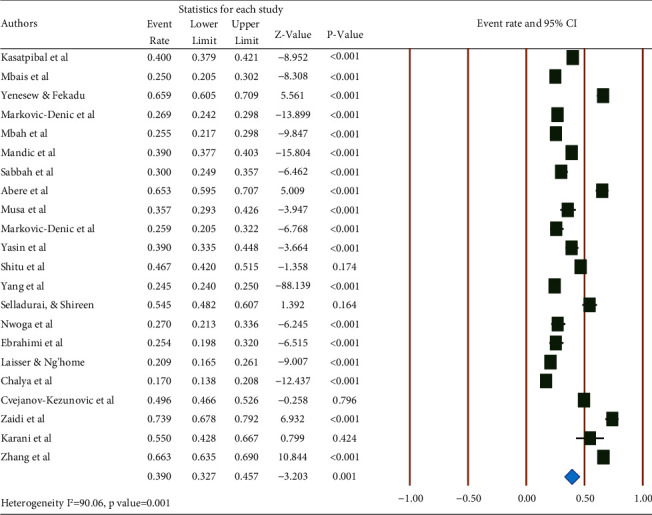
Pooled prevalence of occupational exposure to blood and body fluids in the last 12 months among healthcare workers.

The last year's prevalence of occupational exposure to blood and body fluids among healthcare workers was found to be 39.0% (95% CI: 32.7, 45.7) with a *P*-value of <0.001 (Figure 2).

**Figure 3:**
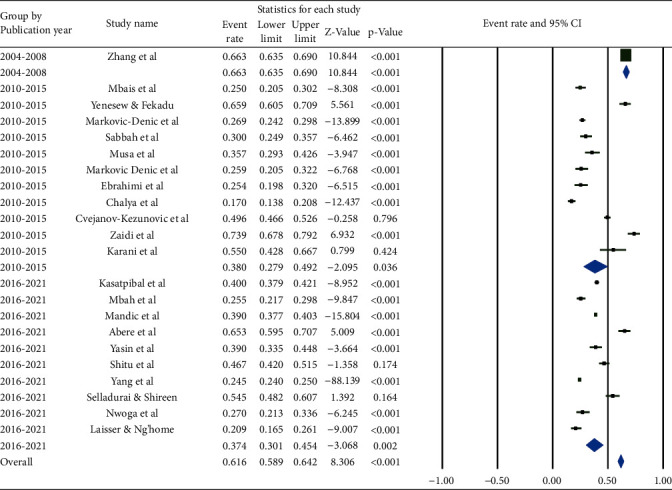
Pooled prevalence of occupational exposure to blood and body fluids in last 12 months among healthcare workers based on the publication year.

Based on a subgroup analysis by publication year, there was a relatively equal prevalence of BBFs in the last 12 months that accounted for 38.0% (95% CI: 27.9, 49.2%) and 37.4% (95% CI: 30.1, 45.4%) for those articles published between 2010 and 2015 and 2016 and 2021, respectively (Figure 3).

**Figure 4:**
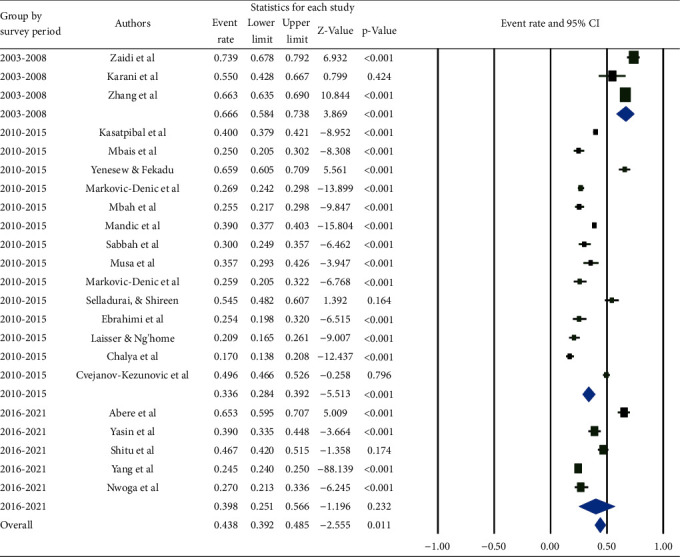
Pooled prevalence of occupational exposure to blood and body fluids in last 12 months among healthcare workers based on the survey period.

According to a subgroup analysis by survey year, studies conducted between 2003 and 2008 had the highest pooled prevalence (66.6% (95% CI: 58.4, 73.8%)), while studies conducted between 2010 and 2015 had the lowest (33.6% (95% CI: 28.4%, 39.2%)) (Figure 4).

**Figure 5:**
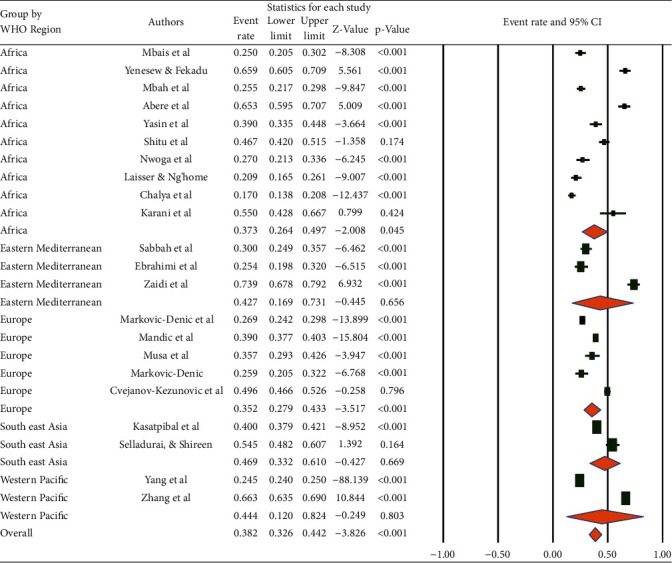
Prevalence of occupational exposure to blood and body fluids in the last 12 months among healthcare workers based on WHO regions.

Based on the WHO regions, the highest prevalence of last year's BBF was observed in the Southeast Asia Region (46.9% (95% CI: 33.2, 61.0%)) followed by the Western Pacific (44.4% (95% CI: 12.0, 82.4%)). The lowest prevalence was reported from the European Region (35.2% (95% CI: 27.9, 43.3%)) (Figure 5).

#### 3.3.2. Career Time Prevalence of Exposure to BBFs

**Figure 6:**
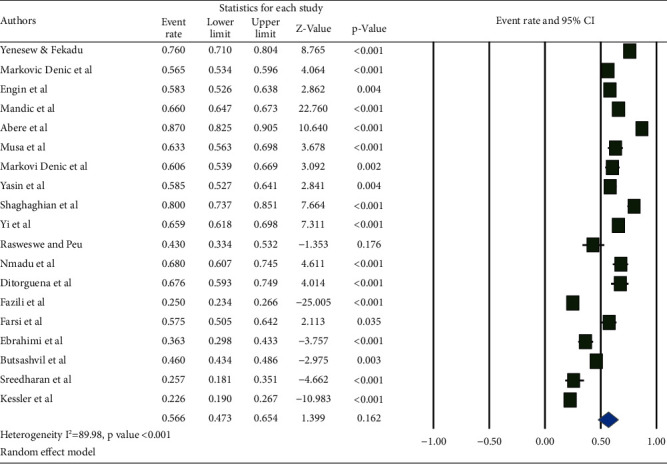
Overall pooled prevalence of occupational exposure to blood and body fluids in career time among healthcare workers.

The career time prevalence of occupational exposure to blood and body fluids among healthcare workers was found to be 56.6% (95% CI: 47.3, 65.4) (Figure 6).

**Figure 7:**
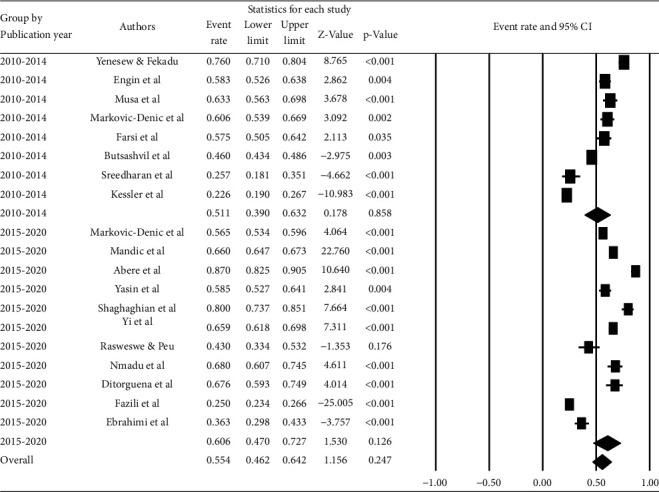
Pooled prevalence of occupational exposure to blood and body fluids in career time among healthcare workers based on the publication year.

Based on a subgroup analysis by publication year, the highest career time pooled prevalence (60.6% (95% CI: 47.0, 72.7%)) was reported among the studies published from 2015 to 2020, while the lowest prevalence (51.1% (95% CI: 39.0, 63.2%)) was reported among the studies published from 2010–2014 (Figure 7).

**Figure 8:**
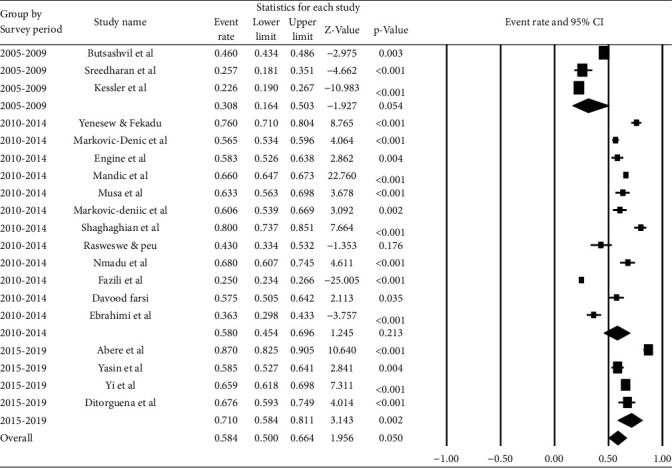
Pooled prevalence of career time occupational exposure to blood and body fluids among healthcare workers based on the survey period.

Based on the survey period, the highest career time pooled prevalence (71.0% (95% CI: 58.4, 81.1%)) was reported in the study conducted from 2015 to 2019, while the lowest prevalence (30.8% (95% CI: 16.4, 50.3%)) was reported among the study published from 2005 to 2009 (Figure 8).

**Figure 9:**
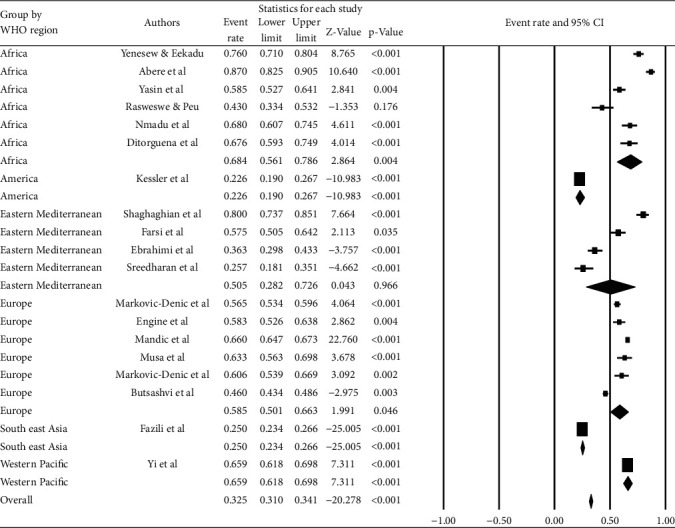
Prevalence of career time occupational exposure to blood and body fluids among healthcare workers based on WHO regions.

Based on the WHO regions, the African region had the highest prevalence (68.4% (95% CI: 56.1, 78.6%)) of occupational exposure to BBFs, followed by the Western Pacific (65.9% (95% CI: 61.8, 69.8%)). The American Region had the lowest prevalence (22.6% (95% CI: 19.0, 26.7%)) (Figure 9).

### 3.4. Sensitivity Analysis Results

The sensitivity analysis was conducted by dropping small sample size and large sample size. However, there was no significant change found in the prevalence of both career time and last year occupational exposure to blood and body fluids (Table 2).

## 4. Discussion

A total of 3912 studies were retrieved from selected electronic databases, of which 1430 duplicate articles were excluded. A total of 33 studies conducted on 54328 HCWs from 2003 to 2021 were included in the systematic review and meta-analysis. Direct comparison of the current findings with other findings was difficult because of a lack of similar systematic reviews and meta-analyses. The authors found only one systematic review and meta-analysis conducted to determine occupational exposure to BBFs among HCWs in Africa. However, we considered other occupational-related injuries or exposures, such as percutaneous injuries and needlestick injuries.

In the workplace, blood and body fluids are a major risk factor for the transmission of various blood-borne infections to healthcare workers [49] such as hepatitis B virus, hepatitis C virus, and human immunodeficiency virus, the three leading causes of occupationally related blood-borne infections among HCWs [50]. However, this study found that the last year's prevalence of occupational exposure to blood and body fluids among healthcare workers accounted for 39.0% (95% CI: 32.7, 45.7). The current study found a lower prevalence of BBFs than another study conducted in 21 African countries, which discovered 48.0% prevalence [6].

Other studies conducted in Africa reported a one-year prevalence rate of blood exposure accounted for 84.0% [51], which was higher than the current finding. The variation may be related to the scope of the study because the current study included studies conducted in both developing and developed countries. Occupational exposure to hazards continues to be a public health concern globally. Another study found that about 36.4% (95% CI: 32.9–40.0) of HCWs were exposed to percutaneous injuries in the previous year, which is lower than the current finding. The variation could be due to differences in the outcomes of these studies because HCWs can be exposed to blood and other body fluids in different ways, such as needlestick injuries or contact with contaminated objects or mucous membranes.

Similarly, this study found that the prevalence of BBF exposure in the last year in the Africa Region was 37.3% (95% CI: 26.4, 49.7), which was in line with the finding of another study, which reported about 48.0% prevalence of exposure [6]. Furthermore, this study found a variation in the prevalence of BBFs among different regions of the world. For example, the highest last 12-month prevalence of BBF exposure was reported from the Southeast Asia Region (46.9% (95% CI: 33.2, 61.0%)), while the lowest prevalence was observed in the study conducted in the European Region (22.6% (95% CI: 19.0, 26.7%). The variation may be related to the difference in implementation of health and safety guidelines or standard precautions or differences in the healthcare system.

On the other hand, this study found a career time prevalence of occupational exposure to blood and body fluids among healthcare workers accounted for 56.6% (95% CI: 47.3, 65.4). This finding was lower than the finding of another study conducted in African countries that found 65.7% (95% CI: 59.7–71.6) prevalence of BBFs [6]. The variation may be related to the included region in the study because this study found the career time prevalence of BBF exposure among HCWs in the African region accounted for 68.4% (95% CI: 56.1, 78.6), which was in line with the finding of another study, which found 65.7% [6].

Furthermore, more than half of the HCWs in the African Region, Western Pacific and Pacific, and European Region were exposed to BBFs. The high prevalence could be due to inadequate healthcare systems and poor occupational health and safety practices. Additionally, even though the highest prevalence was observed in the African region, the study found an increase in the career time prevalence of BBF exposure from 2005 to 2020. This indicates that there is a high risk of being to be exposed to blood-borne diseases among HCWs.

Overall, the study found a high prevalence of occupational exposure to BBFs in the last year (more than one in three HCWs) and throughout the career time (more than two in three HCWs) among healthcare workers. However, exposure to blood and body fluids has serious health implications because exposure to blood and other body fluids is the potential source of blood-borne pathogens such as HBV and HIV that need critical attention to protect the workers' health.

Therefore, applying standard precautions, occupational health and safety measures or services, regular training on infection prevention, and proper implementation of guidelines play a major role in reducing BBFs and preventing infectious diseases in the healthcare system.

### 4.1. Possible Prevention or Control Strategies

Integrated approaches to occupational health and safety, including engineering measures, administrative policy, and the use of personal protective equipment, should be implemented to control, eliminate, or reduce occupational exposure to hazards [52], including BBFs. Furthermore, there is a need to implement priority strategies, which include strengthening of international and national policies for health at work, promotion of a healthy work environment, healthy work practices, strengthening occupational health services, development of occupational health standards, and strengthening of research [53].

### 4.2. Limitations of the Current Study

There was an unequal distribution of occupations among the included articles that make the comparison of BBFs exposure among different occupations more difficult. On the other hand, the prevalence of occupational exposure to BBFs in some regions was not covered due to the lack of studies in these regions. There were a few studies from developed countries conducted on the outcome of interests.

## 5. Conclusions

This systematic review and meta-analysis found a higher percentage of career time and previous one-year global occupational exposures to blood and body fluids among healthcare workers. The study suggests that more than one in three and two in three healthcare workers were exposed to BBFs annually and in their career time, respectively. Therefore, efforts should be made to reduce the high burden of occupational blood and body fluid exposures through effective implementation of standard precaution measures along with occupational health and safety measures.

## Figures and Tables

**Table 1:** Overall characteristics of articles included in the systematic review and meta-analysis, 2021.

Author	Survey year	Pub. year	*N*	12 months	Lifetime	Participant	Setting	Study design	Country	Socioeco status	Risk of bias
Kasatpibal et al. [40]	2011-2012	2016	2031	40.0	NA	Nurses	Hospital	Cross-sectional	Thailand	Developing	Low
Mbaisi et al. [41]	2010	2013	305	25.0	NA	Doctors, nurses, clinical officers, laboratory personnel, dentists, supportive staff, and students	Hospital	Cross-sectional	Kenya	Developing	Low
Yenesew and Fekadu [19]	2012	2014	317	65.9	76.0	Nurses, health officers, health assistants, medical doctors, laboratory technicians, and dentists	Healthcare facilities	Cross-sectional	Ethiopia	Developing	Low
Markovic-Denic et al. [25]	2012	2015	983	26.9	56.5	Healthcare workers	Hospital	Cross-sectional	Serbia	Transition	Moderate
Mbah et al. [20]	2013	2020	444	25.5	NA	Doctors and nurses	Health center and hospital	Cross-sectional	South Africa	Developing	Low
Engin et al. [42]	2010	2014	300		58.3	Nurses, physicians, cleaning staff, student nurses, and laboratory technicians	Hospital	Cross-sectional	Turkey	Developing	Moderate
Mandić et al. [24]	2013	2018	5247	39.0	66.0	Physician, nurses, laboratory technicians, and support staff such as cleaners and workers in laundry and sterilization	Hospital	Cross-sectional	Serbia	Transition	Low
Sabbah et al. [43]	2011/12	2013	277	30.0	NA	Physician and nurses	Hospital	Cross-sectional	Lebanon	Developing	Low
Abere et al. [17]	2018	2020	277	65.3	87.0	Nurse, medical doctor, laboratory technology, health officer, midwife, pharmacy	Hospital	Cross-sectional	Ethiopia	Developing	Low
Musa et al. [44]	2013	2014	196	35.7	63.3	Physicians and nurses/technicians	Hospital	Cross-sectional	Bosnia and Herzegovina	Transition	Low
Marković-Denić et al. [23]	2011	2013	216	25.9	60.6	Nurses and doctors	Hospital	Cross-sectional	Serbia	Transition	Moderate
Yasin et al. [2]	2017	2019	282	39.0	58.5	Nurse, laboratory, medical doctor, midwife, and others	Hospital	Cross-sectional	Ethiopia	Developing	Low
Shaghaghian et al. [27]	2011	2015	191		80.0	Dental students	Dental school department	Cross-sectional	Iran	Developing	Low
Yi et al. [29]	2015	2018	548		65.9	Nurses	Hospital	Cross-sectional	China	Developing	Low
Rasweswe and Peu [22]	2014	2020	94		43.0	Nurses	Hospital	Cross-sectional	South Africa	Developing	Moderate
Nmadu et al. [38]	2011	2016	172		68.0	Nurses, midwives, CHOs, CHEWs, laboratory technicians, pharmacy technicians, and ward attendants	Primary healthcare centers	Cross-sectional	Nigeria	Developing	Low
Shitu et al. [18]	2020	2021	424	46.7	NA	Midwives	Hospitals and health centers	Cross-sectional	Ethiopia	Developing	Low
Yang et al. [30]	2019	2021	33,156	24.5	NA	Doctors, nurses, anesthetists, midwives, laboratory personnel, and others	Hospital	Cross-sectional	China	Developing	Moderate
Ditorguena et al. [45]	2018	2019	136		67.6	Doctors, surgeons, nurses, midwives, laboratory technicians, and nursing assistants	Hospital	Cross-sectional	Togo	Developing	Moderate
Fazili et al. [34]	2014	2017	2763		25.0	Doctors, nursing staff, lab staff, sanitation staff, administration, laundry, and linen	Tertiary care institute	Cross-sectional	India	Developing	Moderate
Farsi et al. [28]	2010	2012	200		57.5	Physicians, residents, medical interns, nurses, laboratory personnel, housekeepers, cleaners, and others	Hospital	Cross-sectional	Iran	Developing	Low
Selladurai and Shireen [35]	2014	2019	240	54.5	NA	Nurses, laboratory, technicians, interns, and resident doctors	Hospital	Cross-sectional	India	Developing	Moderate
Nwoga et al. [39]	2018	2020	200	27.0	NA	Nurse, laboratory scientist/technician, and others		Cross-sectional	Nigeria	Developing	Low
Ebrahimi et al. [26]	2010	2015	193	25.4	36.3	Laboratory personnel	Hospital	Cross-sectional	Iran	Developing	Moderate
Laisser and Ng'home [32]	2015	2017	277	20.9	NA	Doctors, clinical officers, nurses, laboratory personnel, mortuary attendants, and housekeeping staff	Health facilities	Cross-sectional	Tanzania	Developing	Low
Chalya et al. [33]	2013-14	2015	436	17.0		Doctors, nurses, laboratory staff, and auxiliary health workers	Hospital	Cross-sectional	Tanzania	Developing	Low
Butsashvili et al. [46]	2006-07	2012	1386		46.0	Physician and nurse	Hospitals	Cross-sectional	Georgia	Transition	Low
Cvejanov-Kezunović et al. [47]	2011	2014	1043	49.6	NA	Physicians, nurses, lab personnel, and other non-HCW (cleaning, delivery, and maintenance)	Hospital	Cross-sectional	Croatia	Developed	Low
Zaidi et al. [36]	2008	2012	230	7.39	NA	Nurses, physician, lab staff, and other healthcare providers	Hospital	Cross-sectional	United Arab Emirates	Developing	Low
Sreedharan et al. [37]	2009	2010	101	NA	25.7	Nurses	Hospital	Cross-sectional	United Arab Emirates	Developing	Moderate
Karani et al. [21]	2008	2011	64	55	NA	Medical interns	Hospital	Cross-sectional	South Africa		Moderate
Kessler et al. [48]	2007	2011	455	NA	22.6	Medical residents, emergency residents, nursing, and dental professional	Not specified	Cross-sectional	USA	Developed	Low
Zhang et al. [31]	2003/4	2009	1144	66.34	NA	Physician, nurse, and laboratory technician	Hospital	Cross-sectional	China	Developing	Low

**Table 2:** Sensitivity analysis by dropping small sample size and large sample size.

Criteria	Initial prevalence	After analysis (%)	Heterogeneity	95% CI
Dropping 2 small sample size (career time)	56.6% [95% CI: 47.3, 65.4]	58.2	99.137	47.6, 68.1%
Dropping one smallest sample size (career time)	56.6% [95% CI: 47.3, 65.4]	56.4	99.095	46.0, 66.2%
Dropping large sample size (career time)	56.6% [95% CI: 47.3, 65.4]	55.1	98.649	44.7, 65.1%
Dropping small sample size (last year)	39.0% (95% CI: 32.7, 45.7)	38.3	99.088	32.0, 45.1%
Dropping large sample size (last year)	39.0% (95% CI: 32.7, 45.7)	39.8	97.843	33.9, 46.0%

## Data Availability

Almost all data are included in this study. Some additional data will be available from the corresponding author upon reasonable request.

## Abbreviations

BBF: Blood and body fluids
CMA: Comprehensive meta-analysis
HBV: Hepatitis B virus
HCV: Hepatitis C virus
HIV: Human immune-deficiency virus
HCW: Healthcare workers
JBI: Joanna Briggs Institute
PRISMA: Preferred Reporting Items for Systematic Review and Meta-Analysis
WHO: World Health Organization
MeSH: Medical Subject Heading.
